# SHD reviewers January 2024–December 2024

**DOI:** 10.1093/skinhd/vzaf035

**Published:** 2025-05-12

Successful and reliable publishing is dependent on expert, objective and timely peer review. The editorial team greatly appreciates the goodwill, time and effort that goes into writing these reviews, and I would like to thank all those who reviewed for the SHD last year.

Al-Muriesh, Maher

Abbasi, Mohammed

Abdel Azim, Amira

Abdel Naser, Mohamed Badawy

Abdelhamed, Amr

Abdelmaksoud, Ayman

Abdelrahman, Islam Fathy Soliman

Abdo, Hamed

Abu El-Hamd, Mohammed

Affleck, Andrew

Afzal, Usamah

Agak, George

Agarwal, Puneet

Agarwal, Shipra

Ajani, Atinuke

Akasaka, Eijiro

Akhras, Victoria

Akuffo-Addo, Edgar

Alfonso, Jose Hernan

Alhomida, Faris

Ali, Atif

Alkhayal, Fares

Allsop, Daniel

Álvarez-Salafranca, Marcial

Alzahrani, Ahmad

Andersen, Klaus

Andersen, Monica

Angus, Janet

Ansari, Farzana

Arents, Bernd

Aristizabal, Miguel

Aromolo, Italo Francesco

Ashique, Karalikkattil

Asilsoy, Suna

Attia, Enas

Atzori, Laura

Aung, Tim

Aw, Derrick

Azad, Jaskiran

Bagatin, Edileia

Balieva, Flora

Banila, Cristiana

Barlow, Richard

Basmanav, F. Buket

Baumrucker, Steven J.

Beausillon, Chrstopher

Beazley, Ryan

Begolka, Wendy

Beksac, Burcu

Berna Rico, Emilio

Bighetti, Stefano

Bishnoi, Anuradha

Boch, Katharina

Boekema, Bouke

Boey, Justin Jia Jun

Boffano, Paolo

Borlu, Murat

Boulanger, N.

Bradshaw, Sarah

Brownell, Isaac

Bueno, Juan

Buhl, Timo

Bunker, Christopher

Buontempo, Michael

Burge, Russel

Burgess, Gary

Butt, Sundas

Byrne, Berbie

Caesar, Jenny

Calzavara-Pinton, Pier Giacomo

Cansu, Dondu

Caplan, Avrom

Capozza, Korey

Cappilli, Simone

Cardenas-de la Garza, Jesus Alberto

Carmichael, Andrew

Cazzaniga, Simone

Chan, Kok-Gan

Chanal, Johan

Chandler, David

Chang, Kuan

Chaudhuri, Ratan K.

Chen, Jinghan

Chen, Yi An

Chernyshov, Pavel

Cherry, Lindsey

Chiner, Eusebi

Chiramel, Minu

Chiu, Yvonne

Cho, Soo ick

Cho, Yung-Tsu

Choi, Ellie

Choudhary, Cherry

Ciccarese, Giulia

Clarke, Elaine

Collgros, Helena

Connolly, Aveen

Corazza, Monica

Coulombe, Jerome

Criado, Paulo

Cronhjort, Samuel

Damiani, Giovanni

Das, Kinnor

Dassoni, Frederica

de Andrade, Heitor, Jr

de Brito, Marianne

de Souza, Melanie Miyanji

de Toro, Gonzalo

de Troya-Martin, Magdalena

Del Pozzo-Magana, Blanca

Demetriou, M.

Di Stefani, Alessandro

Diaz Calvillo, Pablo

Doering, Nicola

Dogra, Sunil

Dolianitis, Con

Dubash, Faisal

Dubois, Anna

Duffy, Jonathan

Dumic, Igor

Edwards, Harrison

Eid, Amira

Emelianov, Vladimir

Estrach, Teresa

Farista, Arshi

Farris, Patricia

Fearfield, Louise

Feldman, Steven

Fernandes, Neil

Fernández Peñas, Pablo

Fong, Kenneth

Fujita, Yasuyuki

Fujiwara, Hiroshi

Furukawa, Fukumi

Furuta, Junichi

Gallais Sérézal, Irène

Galvan Casas, Cristina

Gao, Xinghua

Gardner, Louis

Gawkrodger, David

Geh, Jenny

Geller, Shamir

George, Leni

Ghio, Daniela

Giavedoni, Priscila

Godor, Dorottya

Goebeler, Matthias

Goldenberg, Michael

Gong, Yu

Gönülal, Melis

Goutos, I.

Grantham, Henry

Greenberger, Shoshana

Greenblatt, Danielle

Grijsen, Marlous

Gross, Kenneth

Guimaraes, F.

Gulanikar, Anirudha

Guo, Zongsheng

Hajdu, Krisztina

Hamdy, Nadia M.

Hampton, Philip

Hanafy, Noha Sami

Haneke, Eckart

Harris, Charlotte

Harry, Tubonye

Harvima, Ilkka

Haselgruber, Sofía

Hashimoto, Takashi

Hashizume, Hideo

Hassan, Hafidh

Hassib, Nehal F.

Hata, Masaharu

Ha-Wissel, Linh

Hayashi, Ryota

Heelan, Kara

Heerfordt, Ida M.

Hefez, Louise

Hieta, Niina

Honnor, Kate

Hrin, Matthew

Hughes, Jenny

Hussain, Walayat

Ibrahim, Fahad

Inoue, Takamitsu

Ishitsuka, Yosuke

Jain, Manu

Janda, Monika

Jayasinghe, Dilki

Jerkovic Gulin, Sandra

Jiang, Hao

Junejo, Muhammad

Kapoor, Krishan Mohan

Kar, Bikash

Karanovic, Sanja

Kaul, Subuhi

Kavaklieva, Svetlana

Kempf, Werner

Kennedy, Jake

Khair, Tabassum

Khan, Farishta

King-Stokes, Natalie

Kirby, Lisa

Kishibe, Mari

Kitayama, Shohei

Koc Yildirim, Sema

Koh, Xuan Qi

Kono, Michihiro

Kostik, Mikhail M.

Kotb, Iman

Kounidas, Georgios

Kravvas, Georgios

Kuraishi, Yasushi

Laheru, Dhruv

Lambert, Jo

Langan, Ewan

Laws, Philip

Lawton, Sandra

Leahy, Marion

Lee, Ava

Lee, Haur Yueh

Lee, Kachiu

Licata, Gaetano

Lim, Chris

Lin, Zhimiao

Lingam, Claire

Liu, Bo

Liu, Yale

Liu-Smith, Feng

Llamas Molina, Jose Maria

Long, Valencia

Longo, Caterina

Malik, Bilal Haider

Martin, Arthur

Martin-Gorgojo, Alejandro

Martins, Giselle

Matsuda, Kazuki

Mauskar, Melissa

McCourt, Collette

McPherson, Tess

Megna, Matteo

Melgosa Ramos, Francisco Javier

Menezes, Antonio S., Jr

Minakawa, Satoko

Misery, Laurent

Miteva, Mariya

Miura, Hiroyuki

Miyagaki, Tomomitsu

Molina-Leyva, Alejandro

Molloy, Kevin

Montero-Vilchez, Trinidad

Moss, Celia

Muir, Jim

Mun, Andrey

Muñoz-Barba, Daniel

Muralidharan, Vijaytha

Myskowski, Patricia

Narang, Tarun

Nardone, Beatrice

Natsuga, Ken

Navarrete-Dechent, Cristian

Navarro-Triviño, Francisco José

Nazzaro, Gianluca

Neyns, Bart

Ng, Chau Yee

Nicolau, Rafaela

Nielsen, Kari

Nikookam, Yasmin

Nomura, Toshifumi

Novakovic, Ljuba

Nylander, Elisabet

Ogboli, Malobi

Oh, Byung Ho

Ohn, Jungyoon

Ohyama, Katsuhiro

Oldham, Jaimie

Olivares-Guerrero, Maria

O’Toole, Edel

Owczarczyk-Saczonek, Agnieszka

Owen, Joshua

Ozturk Durmaz, Emel

Pagliari, Carla

Palanivel, Janani

Pan, Meng

Papanikolaou, Marieta

Papathomas, Thomas

Parkins, Greg

Pasquali, Paola

Patel, Tejesh

Patsatsi, Aikaterini

Paudel, Vikash

Peng, Hsien-Li Peter

Pennock, Jo

Pham, James

Podder, Indrashis

Poddighe, Dimitri

Polo Silveira, Luísa

Pudasaini, Prajwal

Quiñones-Vico, Maria Isabel

Rademaker, Marius

Ragamin, Aviël

Rageh, Mahmoud

Rajan M., Bandhala

Rajan, Neil

Recke, Andreas

Reich, Adam

Reikvam, Hakon

Ribero, Simone

Ridd, Matthew

Ridha-Salman, Hayder

Rittie, Laure

Rokia, Maged

Ross, Kehinde

Saad, Sameh

Sanabria de la Torre, Raquel

Sanader Vucemilovic, Ana

Sanchez-Diaz, Manuel

Santer, Miriam

Sasaki, Yasushi

Schettini, Natale

Schmalwieser, Alois

Scott, Sonya

Searle, Tamara

Sechi, Andrea

Selk, Amanda

Senkal, Emine

Sharma, Saraswoti

Shimanovich, Iakov

Shipman, Alexa

Siddig, Emmanuel E.

Sikora, Mariusz

Sil, Abheek

Slominski, Andrzej

Smak Gregoor, Anna

Smit, Amelia

Solomon, Mark

Song, Sheng

Soto-Moreno, Alberto

Sotto, Mirian

Soutou, Boutros

Sperling, Leonard

Steijlen, Olivia

Sterling, Jane

Sticherling, Michael

Stout, Molly

Streadmann, Jessica

Svendsen, Mathias  Tiedemann

Szczecinska, Weronika

Taieb, Alain

Takwale, Anita

Talaganis, John

Tan, Cher-Han

Tan, Zhao

Tang, Edwina

Tang, Keyun

Tawfik, Soha

Temiz, Selami Aykut

Thandar, Yasmeen

Theocharopoulos, Ioannis

Thibau, Isabelle

Tobin, Desmond

Todd, Gail

Totty, Joshua

Trave, Ilaria

Trindade, Maria A.B.

Tso, Simon

Ubago-Rodríguez, Ana

Ugwu, Nelson

Urena-Paniego,  Clara Amanda

Valenzuela, Fernando

Vardomael, Isabelle

Vavassori, Andrea

Vorobyev, Artem

Wainman, Hannah

Walsh, Sarah

Wang, Hui

Watson, Nicola

Wharton, Simon

Witkam, Willemijn

Wong, Vidette

Worsley, Peter

Worsnop, Fiona

Wu, Jashin

Wu, Minjuan

Wu, Weiwei

Yanagi, Teruki

Yancheva, Nina

Yang, Chao-Chun

Yang, Jiankang

Yélamos, Oriol

Yiu, Zenas

Yosipovitch, Gil

Zanini, Ludovica

Zaouak, Anissa

Zhang, Jing-Zhan

Zhang, Junfen

Zhang, Kun

Zhang, Wei

Zhang, Xiaoting

Zhang, X.W.

Zhou, Catherine

Zhuang, Kaiwen

Zorbozan, Orcun

